# Cost-effectiveness of perioperative durvalumab plus FLOT for resectable gastric and gastroesophageal junction adenocarcinoma in the United States

**DOI:** 10.3389/fimmu.2026.1712403

**Published:** 2026-02-10

**Authors:** Jiahao Zhang, Caicong You, Wu Fu, Liushi Zheng, Maobai Liu, Na Li

**Affiliations:** 1 Department of Pharmacy, Shengli Clinical Medical College of Fujian Medical University, Fujian Provincial Hospital, Fuzhou University Affiliated Provincial Hospital, Fuzhou, China; 2 Department of Pharmacy, Fujian Medical University Union Hospital, Fuzhou, China; 3 The School of Pharmacy, Fujian Medical University, Fuzhou, China

**Keywords:** cost-effectiveness, durvalumab, gastric cancer, gastroesophageal junction adenocarcinoma, perioperative therapy

## Abstract

**Objective:**

This study evaluated the cost-effectiveness of perioperative durvalumab plus FLOT compared with FLOT alone in patients with resectable gastric and gastroesophageal junction (G/GEJ) adenocarcinoma in the United States.

**Methods:**

We developed a semi-Markov model to evaluate the cost-effectiveness of perioperative durvalumab plus FLOT compared with FLOT alone from the perspective of U.S. healthcare payers. The model utilized a 10-year time horizon with a 4-week cycle length. Clinical efficacy and safety data were primarily derived from the phase III MATTERHORN trial, while transition probabilities for subsequent lines of therapy were extrapolated from the RAINBOW trial. Direct medical costs were estimated using 2025 pricing data from CMS fee schedules and published literature. Health utility values were obtained from previous studies. Both costs and outcomes were discounted at an annual rate of 3%. Deterministic and probabilistic sensitivity analyses were performed to assess the robustness of the results.

**Results:**

Compared with perioperative FLOT alone, the addition of durvalumab provided an additional 0.84 QALY, with an incremental cost of $104,256.12, yielding an ICER of $124,661.87 per QALY gained. Sensitivity analyses indicated that the cost of durvalumab and the utility associated with event-free survival (EFS) were the key drivers of model uncertainty.

**Conclusion:**

From the perspective of U.S. healthcare payers, perioperative durvalumab plus FLOT is a cost-effective strategy compared with FLOT alone for patients with resectable G/GEJ adenocarcinoma.

## 1 Introduction

Gastric and gastroesophageal junction (G/GEJ) adenocarcinoma is the fifth most common malignancy worldwide and the fourth leading cause of cancer-related mortality. In the United States, approximately 26,890 new cases and 10,880 deaths are reported annually, with a 5-year overall survival rate of only 30% across all stages (1, 2). For resectable G/GEJ adenocarcinoma, surgical resection combined with perioperative chemotherapy remains a cornerstone of treatment to improve survival outcomes. The FLOT4-AIO trial demonstrated that perioperative chemotherapy based on the FLOT regimen (fluorouracil, leucovorin, oxaliplatin, and docetaxel) provides significantly superior efficacy compared with conventional regimens and has been established as the current standard of care for these patients (3). Nevertheless, a substantial proportion of patients receiving standard therapy experience disease recurrence within one year after surgery, and the 5-year overall survival remains below 50% (4). These findings highlight the limitations of current treatment strategies in achieving durable survival benefits and underscore the urgent need to explore more effective therapeutic approaches.

Immune checkpoint inhibitors (ICIs) have demonstrated substantial survival benefits across a variety of advanced solid tumors. Durvalumab, a selective anti–PD-L1 monoclonal antibody, blocks the interaction of PD-L1 with PD-1 and CD80, thereby enhancing T cell–mediated antitumor immune responses (5). Its clinical value has already been established in the perioperative management of muscle-invasive bladder cancer (6). More recently, the global phase III, open-label, randomized controlled MATTERHORN trial (NCT04592913) further evaluated the efficacy and safety of durvalumab in combination with FLOT for patients with resectable G/GEJ adenocarcinoma (7). The trial demonstrated that, compared with FLOT alone, durvalumab plus FLOT significantly improved 2-year event-free survival (67.4% vs. 58.5%; HR, 0.71; 95% CI, 0.58–0.86; P < 0.001) and overall survival (75.7% vs. 70.4%). Notably, after 12 months of treatment, the combination regimen markedly reduced the risk of death (HR, 0.67; 95% CI, 0.50–0.90; P = 0.03), while maintaining an acceptable safety profile (7). Based on these favorable clinical outcomes, the U.S. Food and Drug Administration (FDA) approved durvalumab in combination with FLOT on November 25, 2025, for the treatment of patients with resectable G/GEJ adenocarcinoma (8).

Although durvalumab combined with FLOT has shown promising clinical benefits, the introduction of innovative immunotherapies is often accompanied by substantial increases in treatment costs. This not only imposes a considerable financial burden on patients, families, and society but also poses challenges for the optimal allocation of healthcare resources and reimbursement decision-making. Currently, evidence regarding the cost-effectiveness of this combination regimen within the U.S. healthcare system remains limited. Therefore, the present study developed a pharmacoeconomic decision-analytic model from the perspective of the U.S. healthcare payer to systematically evaluate the cost-effectiveness of perioperative durvalumab plus FLOT in resectable G/GEJ adenocarcinoma. The findings aim to provide robust economic evidence to inform decision-making by clinicians, healthcare administrators, and policymakers, and to serve as an important reference for the clinical adoption and broader implementation of this treatment strategy.

## 2 Methods

As this study is entirely based on previous research and publicly available data, it does not include any new research involving human participants or animals by any of the authors, and therefore does not require approval from an independent ethics committee. The study was completed in 2025. The economic analysis adhered to the methodological guidelines established by the Second Panel on Cost-Effectiveness in Health and Medicine, and the findings are reported in accordance with the Consolidated Health Economic Evaluation Reporting Standards (CHEERS) 2022 checklist, detailed in **Supplementary Table S1**.

### 2.1 Patients and interventions

Data for this study were obtained from the MATTERHORN trial (6), a multicenter, open-label, randomized phase III clinical study. Eligible patients were aged ≥18 years, had histologically confirmed resectable G/GEJ adenocarcinoma, were scheduled to undergo curative surgery, and had not received any prior systemic anticancer therapy for the current malignancy. Additional inclusion criteria required a World Health Organization (WHO)/Eastern Cooperative Oncology Group (ECOG) performance status of 0 or 1, adequate organ and bone marrow function, and provision of a tumor tissue sample prior to enrollment. The expected survival time was ≥24 weeks. Patients who met these criteria were randomized 1:1 to receive either FLOT or DFLOT, as described below:

#### 2.1.1 FLOT group

Docetaxel 50 mg/m² intravenous (day 1), oxaliplatin 85 mg/m² intravenous (day 1), leucovorin 200 mg/m² intravenous (day 1), and fluorouracil 2600 mg/m² continuous intravenous infusion over 24 hours (day 1). Each cycle lasted 2 weeks. Patients received two cycles of neoadjuvant therapy, followed by curative resection within 4–8 weeks after the last neoadjuvant cycle (surgery delayed if >8 weeks), and then two additional cycles of adjuvant therapy within 4–12 weeks post-surgery.

#### 2.1.2 DFLOT group

The same FLOT chemotherapy regimen was administered in combination with durvalumab. Durvalumab 1500 mg was given intravenously on day 1 of each cycle, once every 4 weeks. The neoadjuvant and adjuvant FLOT regimens were identical to those in the control arm. Postoperatively, patients continued durvalumab monotherapy as maintenance therapy for 10 additional cycles (every 4 weeks). In total, durvalumab treatment comprised 14 cycles, including 4 cycles concurrent with chemotherapy and 10 cycles of postoperative monotherapy, until confirmed disease progression, recurrence, or unacceptable toxicity.

Because post-progression treatment strategies were not explicitly reported in the MATTERHORN trial, subsequent therapy was informed by external evidence and clinical practice guidelines. Based on the RAINBOW trial and current National Comprehensive Cancer Network (NCCN) guidelines (9), patients experiencing disease progression were assumed to receive one of the recommended second-line treatment options, namely ramucirumab (8 mg/kg on days 1 and 15) plus paclitaxel (80 mg/m² on days 1, 8, and 15 of a 28-day cycle), until further disease progression. Upon subsequent progression, patients were assumed to receive best supportive care followed by a single hospice care episode prior to death. Follow-up schedules were designed according to NCCN guidelines: chest/abdominal/pelvic CT scans every 6 months for 2 years, then annually up to 5 years; history and physical examination (H&P) and complete blood count (CBC) tests every 3 months for 2 years, then every 6 months until death. Body surface area (BSA) was calculated using the Mosteller formula (BSA = [height (cm) × weight (kg)/3600]) (10). Height and weight data were obtained from the U.S. adult anthropometric reference database (11) (**Table 1**).

**Table 1:** Ranges and distributions of parameters used in the model.

		Range			
Variable	Baseline Value	Minimum	Maximum	Source	Distribution
Risk for main adverse events in DFLOT group					
Diarrhea	6.30%	4.73%	7.88%	(7)	Beta
Anemia	5.10%	3.83%	6.38%	(7)	Beta
Neutropenia	40.90%	30.68%	51.13%	(7)	Beta
Thrombocytopenia	5.30%	3.98%	6.63%	(7)	Beta
Risk for main adverse events in FLOT group					
Diarrhea	6.00%	4.50%	7.50%	(7)	Beta
Anemia	5.10%	3.83%	6.38%	(7)	Beta
Neutropenia	44.60%	33.45%	55.75%	(7)	Beta
Thrombocytopenia	6.00%	4.50%	7.50%	(7)	Beta
Utility					
EFS	0.797	0.598	0.996	(26)	Beta
PD	0.577	0.433	0.721	(26)	Beta
Gastrectomy	0.773	0.580	0.966	(40)	Beta
AEs disutility					
Diarrhea	0.247	0.164	0.348	(18)	Beta
Anemia	0.200	0.15	0.25	(19)	Beta
Neutropenia	0.159	0.106	0.226	(20)	Beta
Thrombocytopenia	0.149	0.101	0.209	(21)	Beta
AEs cost per					
Diarrhea	3928.00	2946.00	4910.00	(18)	Gamma
Anemia	528.00	396.00	660.00	(19)	Gamma
Neutropenia	18987.00	14240.25	23733.75	(20)	Gamma
Thrombocytopenia	96238.00	72178.50	120297.50	(21)	Gamma
Drug cost per cycle					
Durvalumab	12787.50	9590.63	15984.38	(14)	Gamma
Fluorouracil	35.40	26.55	44.26	(14)	Gamma
Leucovorin	62.68	47.01	78.35	(14)	Gamma
Oxaliplatin	61.42	46.06	76.77	(14)	Gamma
Docetaxel	75.23	56.42	94.04	(14)	Gamma
Ramucirumab	20179.74	15134.81	25224.68	(14)	Gamma
Paclitaxel	50.50	37.87	63.12	(14)	Gamma
Administration cost per					
CT	171.10	128.33	213.88	(15)	Gamma
Ppps, initial visit	165.44	124.08	206.80	(15)	Gamma
Ppps, subsequent visit	130.15	97.61	162.69	(15)	Gamma
CBC	19.00	14.25	23.75	(22)	Gamma
IV infusion 1h	129.16	96.87	161.45	(15)	Gamma
IV infusion over 1h	156.79	117.59	195.99	(15)	Gamma
Removal of stomach	1910.80	1433.10	2388.49	(15)	Gamma
Best supportive care	16632.03	16955.57	28259.28	(17)	Gamma
Hospice care	4,563.28	3422.46	5704.10	(24)	Gamma
Body weight (kg)	84.70	63.53	105.88	(11)	Normal
BSA (m^2^)	1.99	1.49	2.48	(10)	Normal
Gastrectomy utility duration(cycle)	13.00	6.00	20.00	(16)	Normal
Discount rate, %	3.00	0	5.00	(12)	Fixed

AEs, adverse events; FLOT, fluorouracil + leucovorin + oxaliplatin + docetaxel; DFLOT, durvalumab + FLOT; PD, progressed disease; EFS, event-free survival; BSA, body surface area; Ppps, past pertinent problems; CBC, complete blood count; IV, intravenous.

### 2.2 Model construction

**Figure 1:**
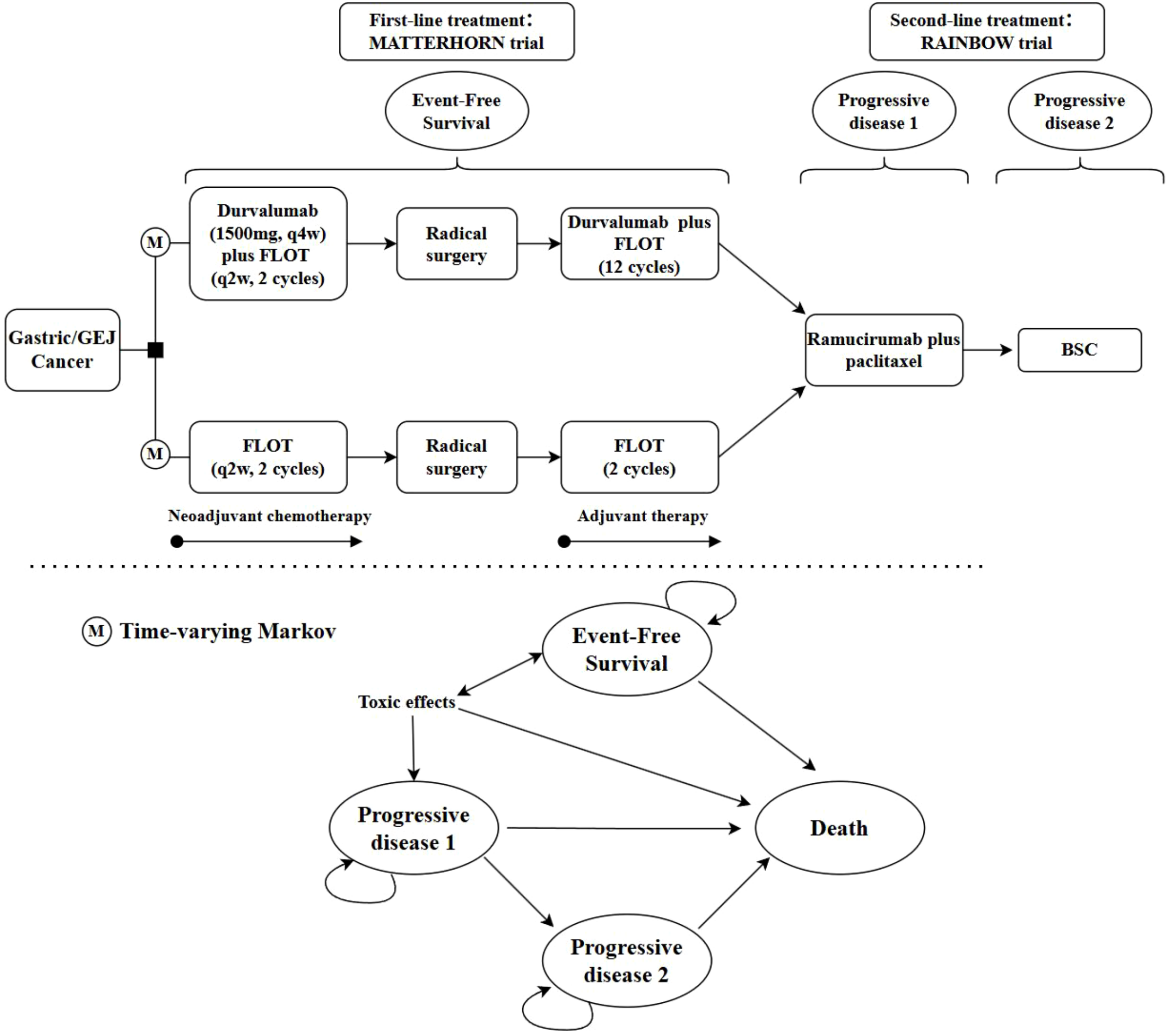
Model structure of a decision tree combining the Markov model with the 4 health states.

A semi-Markov model was developed using R software (version 4.4.3) to evaluate the cost-effectiveness of DFLOT versus FLOT. This framework was chosen to accommodate transitions among postoperative health states—including event-free survival (EFS), recurrence, and death—with state-duration–dependent transition hazards. Conventional Markov models assume time-homogeneous transition probabilities and are therefore limited in representing the non-stationary disease course of resectable G/GEJ adenocarcinoma. In contrast, a time-varying semi-Markov structure permits hazard functions to vary over time, allowing a more faithful representation of postoperative recurrence dynamics and evolving treatment effects. This approach is well established in oncology health-economic modeling and is appropriate for the present analysis. The model adopted a cycle length of 4 weeks and a 10-year time horizon to mitigate uncertainty from long-term extrapolation, as extrapolated survival curves showed that over 95% of patients were deceased by year 10. Four mutually exclusive health states were included: EFS (first-line treatment), first progression (PD1, second-line treatment), subsequent progression (PD2, third-line treatment), and death (**Figure 1**). Transition probabilities from EFS were derived from the MATTERHORN trial (7), while those among PD1, PD2, and death were obtained from the RAINBOW trial (9). Model outcomes included total and category-specific costs, utility values, quality-adjusted life-years (QALY), and the incremental cost-effectiveness ratio (ICER). The willingness-to-pay (WTP) threshold was set at USD 150,000 per QALY for U.S. payers. A half-cycle correction was applied, and both future costs and utilities were discounted at an annual rate of 3% (**Table 1**) (12).

### 2.3 Model parameters

#### 2.3.1 Transition probabilities

Overall survival (OS) and EFS curves were digitized using GetData Graph Digitizer (https://getdata-graph-digitizer.com/index_php.html), and pseudo-individual patient data (IPD) were reconstructed in R software (version 4.4.1) to enable extrapolation of Kaplan–Meier (KM) curves. In accordance with the MATTERHORN trial report, the baseline age at model entry was set to the reported median age for each treatment arm (62 years for DFLOT and 63 years for FLOT) and was used as the starting point for survival extrapolation. For extrapolation, seven commonly used standard parametric distributions (exponential, Weibull, Gompertz, log-normal, log-logistic, gamma, and generalized gamma) and five flexible models (fractional polynomial [FP], restricted cubic spline [RCS], Royston–Parmar [RP], generalized additive model [GAM], and mixture-cure model [MCM]) were fitted. Specifically, both first-order (FP1) and second-order (FP2) forms of the FP model were assessed, while the RP model was fitted on the “odds,” “normal,” and “hazard” scales. The corresponding results are presented in **Supplementary Figures S1**–**S3**. Model selection was based on a comprehensive assessment of visual goodness-of-fit, clinical plausibility, expert opinion, and statistical criteria (AIC and BIC), with preference given to models that most appropriately reflected long-term survival trends (13).

Given the limited maturity of the trial data, some degree of uncertainty was associated with long-term extrapolation. To mitigate the risk of overestimating long-term survival, the baseline analysis prioritized the use of distributions predicting relatively lower survival probabilities among the acceptable models. Specifically, for first-line treatment, the EFS curve of the DFLOT group was fitted using a Weibull distribution, whereas the FLOT group was modeled with an exponential distribution. For second-line treatment, the OS curve was fitted with a Gamma distribution, and the EFS curve was modeled using the RP-hazard model (**Supplementary Figures S5**, **S6**). Finally, time-dependent transition probabilities across health states were derived from the fitted survival curves. Parameter estimates are provided in **Supplementary Table S3**.

#### 2.3.2 Costs estimates

The cost analysis was conducted from the perspective of the U.S. healthcare payer and included only direct medical costs. These comprised drug acquisition costs, laboratory testing, contrast-enhanced abdominal CT scans, blood chemistry (BCB), hospice care, infusion costs per intravenous administration, best supportive care, and management of adverse events (AEs). Only treatment-related adverse events that were both grade ≥3 and had an incidence of at least 5% were included—specifically diarrhea, anemia, neutropenia, and thrombocytopenia—because lower-grade adverse events typically require minimal medical intervention and are expected to have a negligible impact on overall healthcare costs. Drug, infusion, imaging, and laboratory costs were obtained from the Centers for Medicare and Medicaid Services (CMS) and its physician fee schedule (14, 15), while other costs were derived from published literature (16–24). All costs were inflated to 2025 U.S. dollars (25) (**Table 1**).

#### 2.3.3 Utility inputs

As the MATTERHORN trial did not report detailed results from the European Organization for Research and Treatment of Cancer Quality of Life Questionnaire (EORTC QLQ-C30) regarding global health status and quality of life domains, utility values were obtained from previously published studies. The mean health utility values for EFS, disease progression (PD), and death were 0.797, 0.577, and 0, respectively (26). In addition, disutilities associated with grade 3 or higher treatment-related AEs with an incidence of ≥5% were incorporated, with estimates derived from published literature (27, 28). The impact of AEs on quality of life was calculated by multiplying the disutility values by their corresponding incidence rates (**Table 1**).

### 2.4 Sensitivity analyses

To evaluate the robustness of the model results and to identify key drivers of cost-effectiveness, one-way sensitivity analyses (OWSA) and probabilistic sensitivity analyses (PSA) were performed using WTP thresholds of $100,000 and $150,000 per QALY, reflecting commonly applied benchmarks in the United States. In the OWSA, plausible ranges were defined for each parameter. Standard deviations of distributions were obtained from the published literature whenever available; otherwise, a variation of ±25% around the mean value was applied. The results of the one-way sensitivity analysis were illustrated using tornado diagrams to intuitively demonstrate the influence of parameter variation on model outcomes.

To assess the joint impact of parameter uncertainty on model results, a Monte Carlo simulation with 5,000 iterations was performed, during which random sampling was conducted across multiple model parameters. Cost parameters were modeled using gamma distributions, whereas utility and probability parameters were modeled using beta distributions (29). The results of the PSA were presented through scatter plots of cost-effectiveness pairs and cost-effectiveness acceptability curves (CEACs), which displayed the probability of cost-effectiveness of each treatment strategy across a range of WTP thresholds. Furthermore, the expected value of perfect information (EVPI) was calculated based on the PSA results to quantify the economic value of eliminating all parameter uncertainty, thereby informing whether further research is warranted. The baseline values, distributions, and ranges of all model parameters are summarized in **Table 1**.

### 2.5 Scenario analysis

Because the MATTERHORN trial did not report the average number of postoperative durvalumab monotherapy cycles actually completed before recurrence or discontinuation, we conducted a scenario analysis to reflect uncertainty in real-world treatment completion. According to the trial protocol, durvalumab could be administered for up to 10 postoperative monotherapy cycles, and therefore only shortened-cycle scenarios were considered. Based on the reported 52.3% completion rate of adjuvant durvalumab, we assumed that this proportion of patients completed all 10 planned postoperative durvalumab monotherapy cycles, while the initial four perioperative durvalumab cycles administered concurrently with FLOT chemotherapy were held constant. For the remaining 48.3% of patients, reduced postoperative durvalumab monotherapy exposure was modeled, assuming cohort-level average completion of 2, 4, 6, or 8 postoperative cycles.

## 3 Results

### 3.1 Base case analysis

The results of this case analysis are presented in **Table 2**. Over a 10-year time horizon, the total costs were $240,439.92 for the DFLOT group and $134,427.25 for the FLOT group, yielding 3.89 and 3.05 QALY, respectively. Compared with FLOT, DFLOT was associated with an incremental cost of $104,256.12, resulting in an ICER of $124,661.87 per QALY gained. Notably, this ICER falls below the commonly accepted willingness-to-pay threshold of $150,000 per QALY in the United States, indicating that perioperative durvalumab in combination with the FLOT regimen is a cost-effective treatment option for patients with resectable gastric cancer and gastroesophageal junction adenocarcinoma.

**Table 2:** The results of the model’s base-case evaluation.

Parameter	DFLOT	FLOT	Incremental change
Cost ($)			
Drug	240,439.92	134,427.25	106,012.67
AEs Management	13,011.35	14,365.00	-1,353.650
Follow up	2,554.73	2,273.37	281.360
End-of-life	3,004.78	3,689.04	-684.260
Total cost	259,010.78	154,754.66	104,256.12
Total life years	5.61	4.41	1.200
QALY	3.89	3.05	0.840
ICER			124,661.87

FLOT, fluorouracil + leucovorin + oxaliplatin + docetaxel; DFLOT, durvalumab + FLOT; QALY, quality-adjusted life years; ICER, incremental cost-effectiveness ratio.

### 3.2 Sensitivity analysis

**Figure 2:**
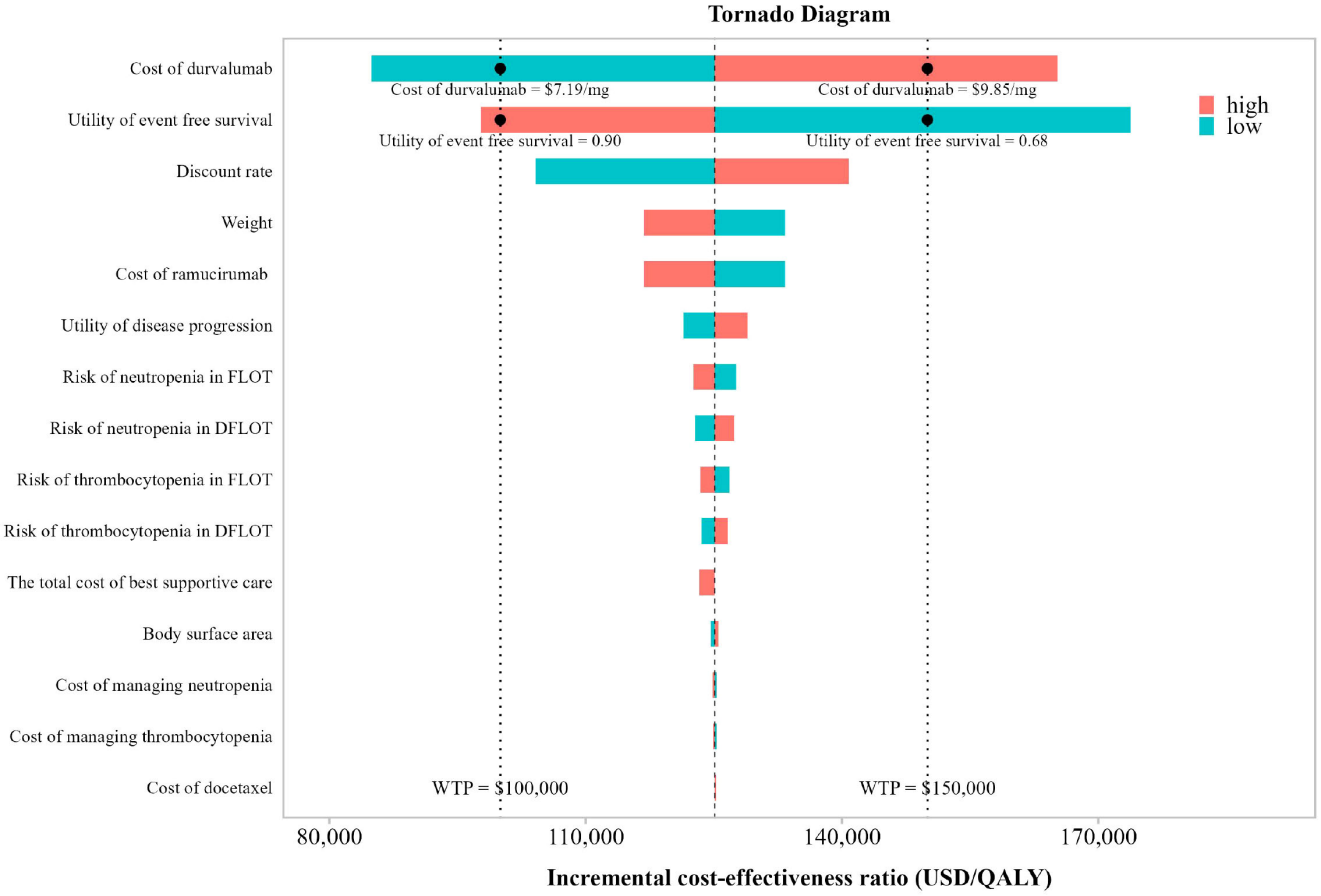
Tornado diagrams illustrating the results.

Results of the OWSA are presented in **Figure 2**. The analysis demonstrated that the cost of durvalumab, the utility associated with EFS and PD, and the discount rate were the most influential parameters affecting the ICER, whereas other parameters exerted moderate or minor effects. When the durvalumab price was varied from $6.43 to $9.85 per g and the EFS utility was adjusted from 0.80 to 0.68, the ICER exceeded the WTP threshold of $150,000 per QALY, indicating that under these conditions the DFLOT regimen would no longer be considered cost-effective. Under a more conservative WTP threshold of $100,000 per QALY, DFLOT was only considered cost-effective when the cost of durvalumab was below $7.19 per mg and the utility of EFS remained below 0.90, highlighting the greater stringency of this threshold.

**Figure 3:**
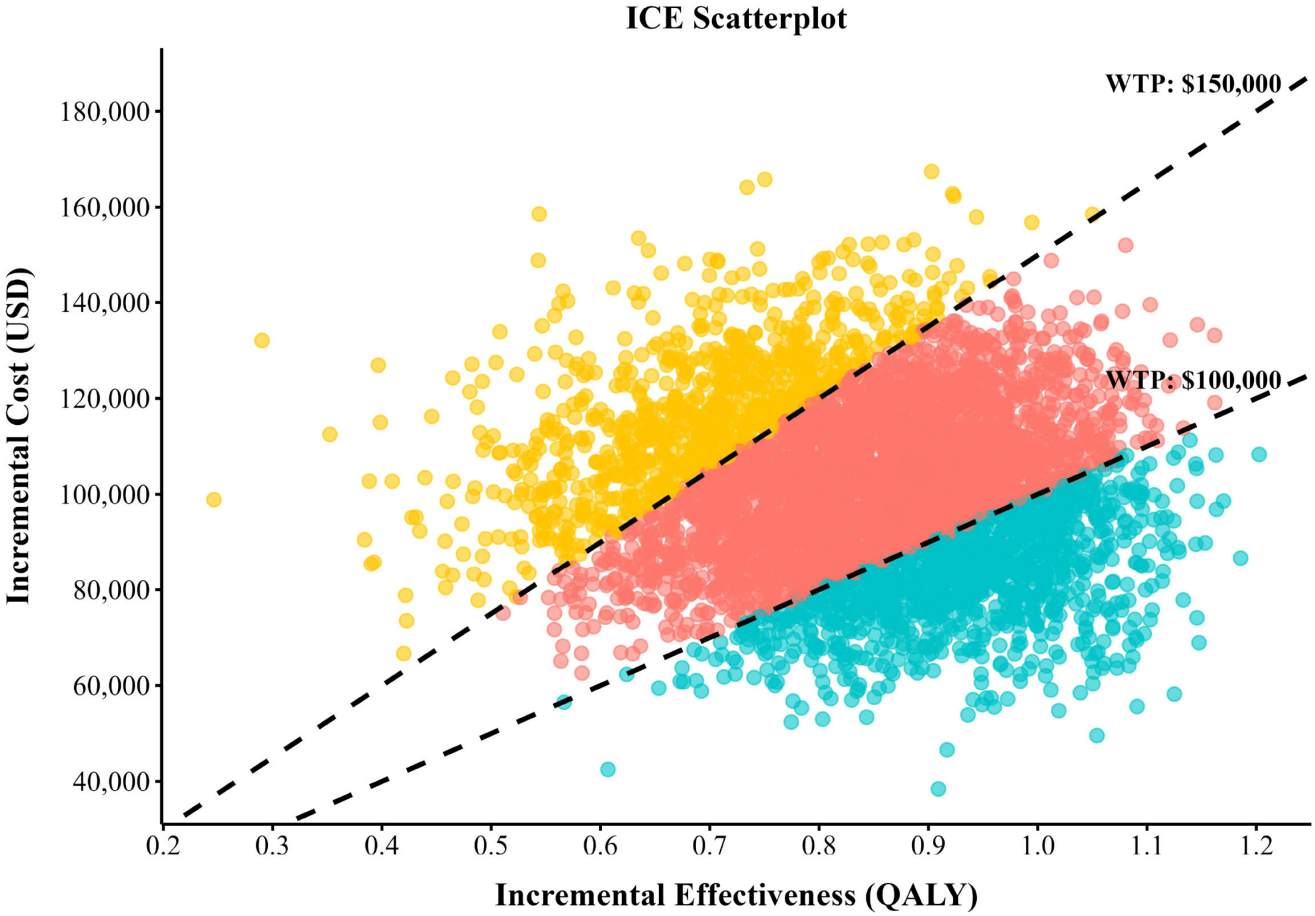
Scatter plot diagrams showing the incremental cost-effectiveness.

**Figure 4:**
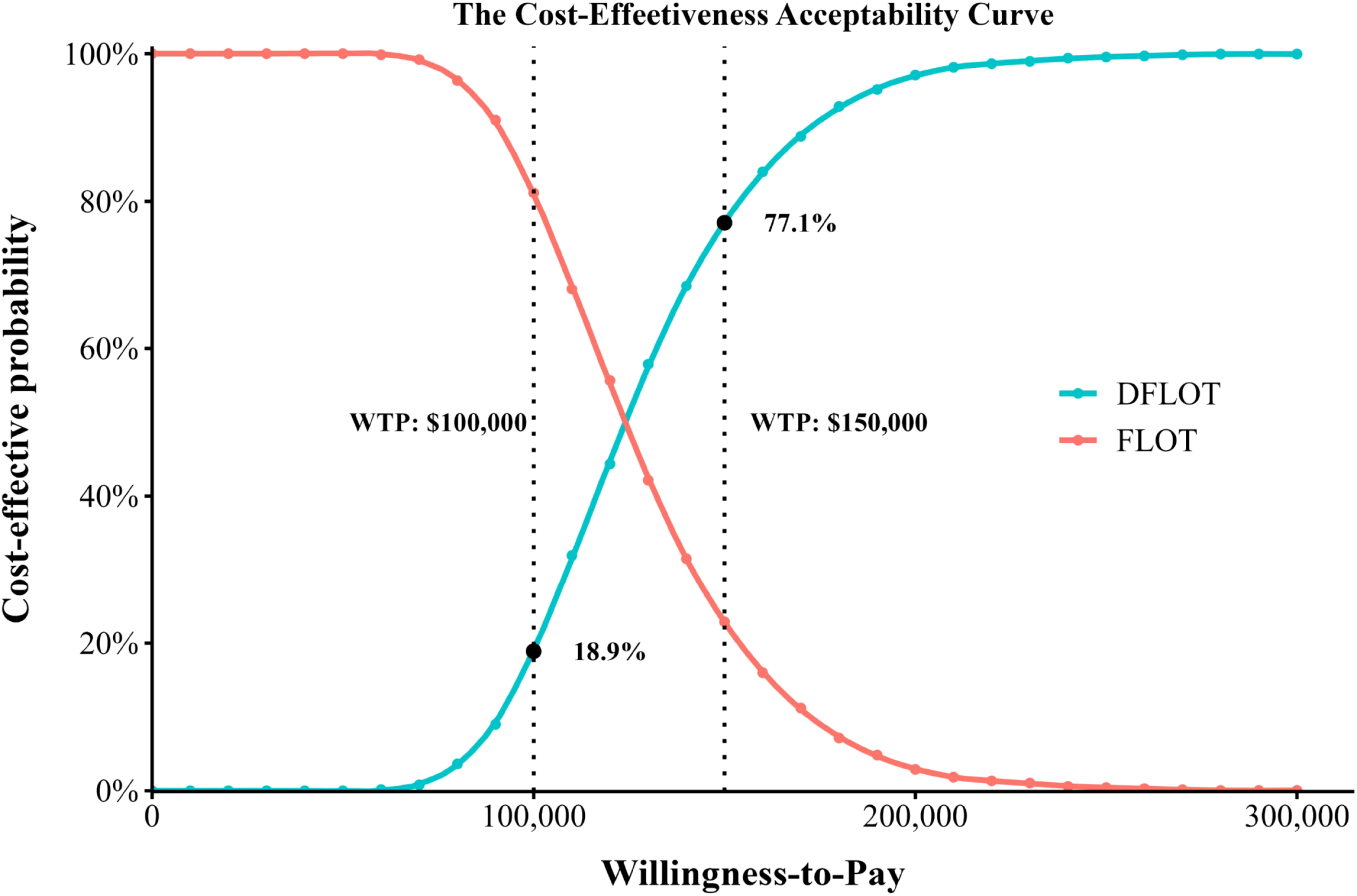
Cost-effectiveness acceptable curve.

Results of PSA are shown in **Figures 3**, **4**. The scatterplot (**Figure 3**) demonstrated that all simulations were above the U.S. per-capita WTP threshold. The cost-effectiveness acceptability curve (**Figure 4**) indicated that at a WTP threshold of $150,000/QALY, DFLOT had a 77.1% probability of being cost-effective. In contrast, when the WTP threshold decreased to $100,000 per QALY, the probability of DFLOT being cost-effective declined substantially to18.85%.

**Figure 5:**
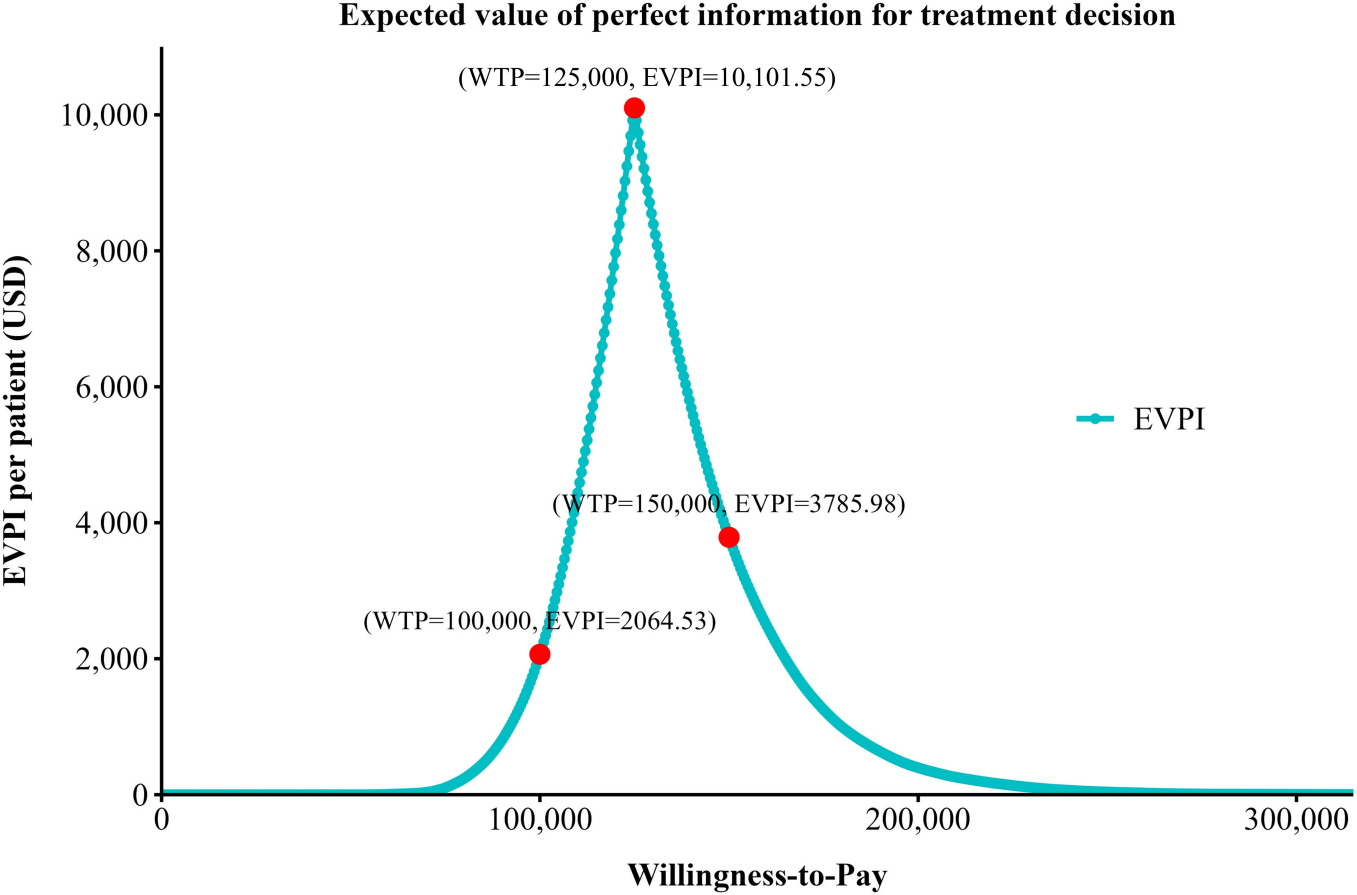
EVPI Curve for treatment decision at varying WTP levels.

Results of the EVPI analysis are depicted in **Figure 5**. The EVPI curve exhibited an inverted V-shaped pattern across different WTP thresholds. The EVPI peaked at a WTP threshold of $125,000 per QALY, reaching $10,101.55 per patient, indicating that decision uncertainty was greatest around this threshold and that the potential economic value of acquiring additional information was maximized at this level. In contrast, at lower and higher WTP thresholds, the EVPI decreased substantially. Specifically, when the WTP threshold was set at $100,000 per QALY and $150,000 per QALY, the EVPI declined to $2,064.53 and $3,785.98 per patient, respectively, suggesting relatively limited decision uncertainty at these thresholds. Furthermore, when the WTP threshold was below $80,000 per QALY or above $200,000 per QALY, the EVPI approached zero, indicating that under these willingness-to-pay levels, the existing evidence was sufficient to support a clear decision and that the marginal value of conducting further research would be minimal.

### 3.3 Scenario analysis

**Supplementary Table S4** shows that reducing the number of postoperative durvalumab cycles substantially decreased the total cost of DFLOT and led to progressively lower ICER values. When 48.3% of patients completed 10, 8, 6, 4, or 2 postoperative cycles, the ICER declined from the base-case value of $124,661.87/QALY to $111,998.19, $98,937.27, $85,458.06, $71,535.96, and $57,140.88/QALY, respectively. Across all reduced-cycle scenarios, DFLOT remained below the $150,000/QALY WTP threshold.

## 4 Discussion

Gastric cancer and gastroesophageal junction adenocarcinoma are highly aggressive and heterogeneous malignancies of the digestive system, with most patients presenting at a locally advanced stage or at high risk of recurrence at the time of diagnosis (30, 31). Perioperative chemotherapy is the current standard of care for resectable G/GEJ adenocarcinoma, among which the FLOT regimen has been demonstrated to significantly improve event-free survival and overall survival (3). Nevertheless, the efficacy of chemotherapy alone remains limited in a subset of patients, with postoperative recurrence and metastasis continuing to pose substantial challenges, and the 5-year overall survival rate remains below 50% (32, 33).

With the growing understanding of gastric cancer heterogeneity, molecular subtypes, and the tumor immune microenvironment, perioperative immunotherapy strategies have emerged as a research focus. Recently, the MATTERHORN trial investigated the potential incorporation of the immune checkpoint inhibitor durvalumab into perioperative treatment. The combination of chemotherapy and immunotherapy not only exerts direct cytotoxic effects on tumor cells but also enhances host immune responses by activating T cell–mediated antitumor immunity, thereby delaying recurrence and improving long-term survival (7). This innovative multimodal approach represents a significant shift in the management of resectable gastric cancer. However, the high cost of immunotherapy has generated considerable debate, as the financial burden of immune checkpoint inhibitors remains a major barrier to their widespread clinical adoption. Previous pharmacoeconomic evaluations in advanced gastric cancer have demonstrated that, although novel regimens provide superior efficacy, their substantially higher costs prevent most from meeting conventional cost-effectiveness thresholds (34, 35). For instance, nivolumab plus chemotherapy as a first-line treatment for locally advanced or metastatic gastric cancer was not considered cost-effective at a willingness-to-pay (WTP) threshold of $150,000/QALY (35). By contrast, perioperative treatment offers a distinct clinical context: patients are at a resectable stage with curative potential, and the long-term benefits of immunotherapy may be more pronounced. Furthermore, reductions in subsequent treatment costs and overall disease management burden suggest that perioperative immunotherapy may hold greater potential for cost-effectiveness from a long-term perspective.

This study employed a time-varying Markov model to evaluate the cost-effectiveness of perioperative durvalumab plus FLOT in patients with resectable G/GEJ adenocarcinoma. Base-case analysis showed that the total costs for the DFLOT and FLOT groups were $240,439.92 and $134,427.25, respectively. The incremental costs were primarily driven by higher treatment expenses, likely attributable to the high price of durvalumab and the longer survival duration associated with the DFLOT regimen. Despite an incremental cost of $106,012.67, DFLOT provided an additional 0.84 QALY compared with FLOT. Moreover, the DFLOT group incurred slightly lower adverse event–related management costs. This difference was primarily driven by higher incidences of grade ≥3 neutropenia and thrombocytopenia in the FLOT arm, both of which are associated with substantial management costs. In contrast, these events occurred less frequently in the DFLOT arm, resulting in lower adverse event–related costs in the model. Overall, the ICER of DFLOT versus FLOT was $124,661.87 per QALY, which falls below the commonly accepted U.S. WTP threshold of $150,000 per QALY. These findings suggest that perioperative durvalumab plus FLOT represents a cost-effective treatment strategy for resectable G/GEJ adenocarcinoma.

From a global perspective, the cost-effectiveness evaluation of ICIs demonstrates that the findings of this study are largely consistent with results observed across multiple tumor types in perioperative or consolidation settings. In non–small cell lung cancer (NSCLC), the PACIFIC trial established durvalumab as consolidation therapy for unresectable stage III NSCLC. However, a recent international cost-effectiveness analysis reported an ICER of approximately $228,788/QALY for durvalumab consolidation in the United States, which substantially exceeded the commonly accepted WTP threshold, indicating a lack of cost-effectiveness in this indication (36). These findings are consistent with the present study, supporting the economic feasibility of perioperative durvalumab plus chemotherapy and highlighting the potential generalizability of this strategy across different tumor types. Notably, compared with treatment of advanced or metastatic disease, immunotherapy in the perioperative or consolidation setting tends to yield superior cost-effectiveness, largely owing to patients’ better baseline health status, more substantial long-term survival benefits, and greater potential for cure or durable disease control (37).

To ensure the robustness of the model, multiple sensitivity analyses were performed. OWSA provided an intuitive illustration of the impact of individual parameter variations on the ICER, while PSA offered a broader perspective by simultaneously accounting for uncertainties across multiple parameters. Within this context, the EVPI further strengthened the robustness of the findings by quantifying the potential opportunity loss associated with decision uncertainty.

Sensitivity analyses were conducted to assess the robustness of the base-case results and to characterize the impact of parameter uncertainty on cost-effectiveness conclusions. The one-way sensitivity analysis identified the acquisition cost of durvalumab as the most influential driver of the ICER, underscoring the central role of drug pricing in determining the economic viability of perioperative immunotherapy. This finding is consistent with the oncology setting, in which the costs of novel agents often dominate total treatment expenditures, and suggests that pricing strategies, reimbursement negotiations, or patient access programs could meaningfully affect the real-world cost-effectiveness of DFLOT. In addition to drug costs, the utility associated with the EFS health state exerted a substantial influence on model outcomes, reflecting the extended time patients are expected to remain in this state. This highlights the importance of accurately capturing health-related quality-of-life benefits in perioperative treatment settings and supports the need for high-quality patient-reported outcome data in future trials of perioperative immunotherapy for gastric and gastroesophageal junction cancers. Probabilistic sensitivity analysis further confirmed the robustness of the base-case findings while demonstrating that the probability of DFLOT being cost-effective was highly sensitive to the WTP threshold. DFLOT showed a high probability of cost-effectiveness at a WTP of $150,000 per QALY, whereas this probability declined substantially under a more conservative threshold of $100,000 per QALY, emphasizing the importance of reporting results across multiple thresholds to facilitate transparent interpretation. The EVPI analysis provided complementary insights into decision uncertainty. The inverted V-shaped EVPI curve, peaking at approximately $125,000 per QALY, indicates that adoption decisions are most finely balanced at intermediate WTP levels. In contrast, EVPI values were markedly lower at both $100,000 and $150,000 per QALY, suggesting limited residual uncertainty and a low expected value of additional research at these thresholds. The choice of WTP threshold reflects a balance among societal preferences, budget constraints, and healthcare priorities. In the absence of an official cost-effectiveness threshold in the United States, contemporary health technology assessment practice—particularly in oncology—commonly adopts a range of $100,000 to $150,000 per QALY (38). Accordingly, we reported results at both thresholds to enhance transparency, while selecting $150,000 per QALY for the base-case analysis in line with prevailing U.S. oncology economic evaluations and the EVPI findings indicating low decision uncertainty at this level (39).

The scenario analysis further demonstrated that uncertainty in postoperative durvalumab completion influences overall costs but does not change the overall economic conclusion. Among the 48.3% of patients who did not complete adjuvant therapy, reducing the assumed number of postoperative cycles from 10 to 2 led to a progressive decrease in the ICER; however, all values remained below the $150,000/QALY threshold. This indicates that the cost-effectiveness of DFLOT remains robust even when postoperative treatment duration varies in real-world practice.

To the best of our knowledge, this is the first cost-effectiveness analysis of durvalumab plus FLOT for resectable gastric and gastroesophageal junction adenocarcinoma conducted in the United States. Nevertheless, several limitations should be acknowledged. First, consistent with most pharmacoeconomic evaluations, adverse event–related costs were estimated based on data from a single clinical trial and were restricted to grade ≥3 adverse events with an incidence of at least 5%. Although lower-grade or less frequent adverse events may incur additional treatment costs or utility losses and thereby introduce some uncertainty, prior studies have suggested that their impact on overall cost-effectiveness outcomes is generally limited. To assess the potential influence of this assumption on model results, we conducted sensitivity analyses in which adverse event incidence rates were varied by ±25%. These analyses demonstrated that such uncertainty did not materially alter the study conclusions. Second, because the MATTERHORN trial has not yet reported health-related quality of life data, the utility values for different health states in this study were primarily derived from previously published literature. Finally, the 10-year long-term survival data in our model were extrapolated from the 5-year short-term follow-up results of the MATTERHORN trial, which may not fully capture real-world disease trajectories.

Despite these limitations, this study provides important preliminary evidence on the cost-effectiveness of durvalumab plus FLOT within the U.S. healthcare system. The findings may offer valuable economic insights for clinicians, healthcare administrators, payers, and policy-makers involved in drug reimbursement and access decisions. Future research should aim to incorporate longer-term clinical follow-up data, real-world quality-of-life assessments, and cost-effectiveness analyses across different patient subgroups to further refine and validate these conclusions.

## 5 Conclusion

From the perspective of a U.S. payer, at a willingness-to-pay threshold of $150,000 per QALY, the addition of durvalumab to FLOT in the perioperative setting improves life expectancy and QALY compared with FLOT alone, representing a cost-effective treatment option for patients with resectable gastric or gastroesophageal junction adenocarcinoma. Future real-world studies and health outcomes assessments are warranted to provide further evidence to guide clinicians, patients, and health insurance policymakers in decision-making.

## Data Availability

The original contributions presented in the study are included in the article/**Supplementary Material**. Further inquiries can be directed to the corresponding author.
