# Supplementary material for: Cost-effectiveness of perioperative durvalumab plus FLOT for resectable gastric and gastroesophageal junction adenocarcinoma in the United States
Source: Front Immunol. 2026 Feb 10;17:1712403. doi: 10.3389/fimmu.2026.1712403 (PMC12930070; doi:10.3389/fimmu.2026.1712403)
Supplement: Supplementary file 1 [file DataSheet1.docx]

**Supplementary Material**

**Table S1**. CHEERS 2022 Checklist.

**Table S2.** AIC and BIC values from each survival model.

**Table S3.** Relevant parameters of survival distribution.

**Table S4.** Scenario analysis results

**Figure S1.** First-line treatment DFOLT survival curve.
**Figure S2.** First-line treatment FOLT survival curve.

**Figure S3.** Estimated best-fitting first-line EFS curves for DFLOT and FLOT.

**Figure S4.** Estimated best-fitting first-line EFS curves for DFLOT and FLOT.

**Figure S5.** Second-line treatment EFS survival curve.

**Figure S6.** Estimated best-fitting for second-line treatment OS and EFS curves

| **Table S1：** CHEERS 2022 Checklist**.** | | |  |
| --- | --- | --- | --- |
| **Topic** | **No.** | **Item** | **Reported** |
| **Title** |  |  |  |
|  | 1 | Identify the study as an economic evaluation and specify the interventions being compared. | Yes |
| **Abstract** |  |  |  |
|  | 2 | Provide a structured summary that highlights context, key methods, results, and alternative analyses. | Yes |
| **Introduction** |  |  |  |
| **Background and objectives** | 3 | Give the context for the study, the study question, and its practical relevance for decision making in policy or practice. | Yes |
| **Methods** |  |  |  |
| **Health economic analysis plan** | 4 | Indicate whether a health economic analysis plan was developed and where available. | Yes |
| **Study population** | 5 | Describe characteristics of the study population (such as age range, demographics, socioeconomic, or clinical characteristics). | Yes |
| **Setting and location** | 6 | Provide relevant contextual information that may influence findings. | Yes |
| **Comparators** | 7 | Describe the interventions or strategies being compared and why chosen. | Yes |
| **Perspective** | 8 | State the perspective(s) adopted by the study and why chosen. | Yes |
| **Time horizon** | 9 | State the time horizon for the study and why appropriate. | Yes |
| **Discount rate** | 10 | Report the discount rate(s) and reason chosen. | Yes |
| **Selection of outcomes** | 11 | Describe what outcomes were used as the measure(s) of benefit(s) and harm(s). | Yes |
| **Measurement of outcomes** | 12 | Describe how outcomes used to capture benefit(s) and harm(s) were measured. | Yes |
| **Valuation of outcomes** | 13 | Describe the population and methods used to measure and value outcomes. | Yes |
| **Measurement and valuation of resources and costs** | 14 | Describe how costs were valued. | Yes |
| **Currency, price date, and conversion** | 15 | Report the dates of the estimated resource quantities and unit costs, plus the currency and year of conversion. | Yes |
| **Rationale and description of model** | 16 | If modelling is used, describe in detail and why used. Report if the model is publicly available and where it can be accessed. | Yes |
| **Analytics and assumptions** | 17 | Describe any methods for analysing or statistically transforming data, any extrapolation methods, and approaches for validating any model used. | Yes |
| **Characterising heterogeneity** | 18 | Describe any methods used for estimating how the results of the study vary for subgroups. | Yes |
| **Characterising distributional effects** | 19 | Describe how impacts are distributed across different individuals or adjustments made to reflect priority populations. | Yes |
| **Characterising uncertainty** | 20 | Describe methods to characterise any sources of uncertainty in the analysis. | Yes |
| **Approach to engagement with patients and others affected by the study** | 21 | Describe any approaches to engage patients or service recipients, the general public, communities, or stakeholders (such as clinicians or payers) in the design of the study. | Yes |
| **Results** |  |  |  |
| **Study parameters** | 22 | Report all analytic inputs (such as values, ranges, references) including uncertainty or distributional assumptions. | Yes |
| **Summary of main results** | 23 | Report the mean values for the main categories of costs and outcomes of interest and summarise them in the most appropriate overall measure. | Yes |
| **Effect of uncertainty** | 24 | Describe how uncertainty about analytic judgments, inputs, or projections affect findings. Report the effect of choice of discount rate and time horizon, if applicable. | Yes |
| **Effect of engagement with patients and others affected by the study** | 25 | Report on any difference patient/service recipient, general public, community, or stakeholder involvement made to the approach or findings of the study | No |
| **Discussion** |  |  |  |
| **Study findings, limitations, generalisability, and current knowledge** | 26 | Report key findings, limitations, ethical or equity considerations not captured, and how these could affect patients, policy, or practice. | Yes |
| **Other relevant information** |  |  |  |
| **Source of funding** | 27 | Describe how the study was funded and any role of the funder in the identification, design, conduct, and reporting of the analysis | Yes |
| **Conflicts of interest** | 28 | Report authors conflicts of interest according to journal or International Committee of Medical Journal Editors requirements. | Yes |

| **Table S2:** AIC and BIC values from each survival model | | | | | | | | |
| --- | --- | --- | --- | --- | --- | --- | --- | --- |
|  | **DFLOT_EFS** | | **FLOT _EFS** | | **SL_EFS** | | **SL _OS** | |
| **Model** | AIC | BIC | AIC | BIC | AIC | BIC | AIC | BIC |
| exp | 177.86 | 182.02 | **211.71** | **215.87** | 190.01 | 193.81 | 174.93 | 178.73 |
| weibull | **174.81** | **183.13** | 212.76 | 221.08 | 158.94 | 166.54 | 136.76 | 144.36 |
| gamma | 176.10 | 184.42 | 213.46 | 221.78 | 146.75 | 154.35 | **133.64** | **141.24** |
| lnorm | 161.98 | 170.30 | 195.16 | 203.49 | 125.58 | 133.18 | 145.95 | 153.55 |
| gompertz | 165.99 | 174.32 | 205.77 | 214.09 | 182.90 | 190.49 | 153.55 | 161.15 |
| llogis | 169.72 | 178.04 | 203.40 | 211.73 | 133.38 | 140.98 | 135.63 | 143.22 |
| gengamma | 158.07 | 170.55 | 194.18 | 206.67 | 127.36 | 138.76 | 135.29 | 146.69 |
| FP1 | 166.01 | 178.50 | 203.75 | 216.24 | 123.52 | 134.91 | 134.21 | 145.60 |
| FP2 | 170.51 | 187.16 | 204.06 | 220.70 | 124.05 | 139.24 | 134.89 | 150.08 |
| RCS | 161.87 | 191.00 | 199.50 | 228.63 | 134.00 | 160.60 | 138.74 | 157.73 |
| RP-hazard | 158.22 | 183.19 | 194.49 | 219.46 | **115.43** | **142.02** | 135.68 | 147.08 |
| RP-odds | 158.17 | 183.14 | 194.56 | 219.53 | 116.36 | 142.95 | 135.36 | 146.76 |
| RP-normal | 158.33 | 170.82 | 198.43 | 223.40 | 125.58 | 133.18 | 134.81 | 146.20 |
| GAM | 161.71 | 190.16 | 199.32 | 227.67 | 147.60 | 162.58 | 138.38 | 155.94 |
| mix-cure | 850.56 | 879.69 | 981.05 | 1010.18 | 142.00 | 153.39 | 493.85 | 505.24 |
| FLOT, fluorouracil + leucovorin + oxaliplatin + docetaxel; DFLOT, durvalumab + FLOT; SL, second line; EFS, event-free survival; AIC, Akaike information criterion; BIC, Bayesian information criterion. | | | | | | | | |

| **Table S3:** Relevant parameters of survival distribution | |
| --- | --- |
| **Variable** | **Value** |
| **Survival model for first-line treatment with DFLOT** | |
| weibull for EFS | shape = –0.14, scale = 1.88 |
| **Survival model for first-line treatment with FLOT** | |
| exp for EFS | parameter = –1.37 |
| **Survival model for Second-line treatment** | |
| RP-hazard for EFS | γ0 = 11.76; γ1 = 6.10; γ2 = 3.71; γ3 = –5.78; γ4 = 4.04; γ5 = –1.11; γ6 = 0.03; knots = 5; scale = hazard |
| Gamma for OS | shape = 0.60; rate = 0.61 |
| FLOT, fluorouracil + leucovorin + oxaliplatin + docetaxel; DFLOT, durvalumab + FLOT; OS, overall survival; EFS, event-free survival. | |

| **Table S4: Scenario analysis results** | | | | |
| --- | --- | --- | --- | --- |
| **Scenario** | **Total Cost** | **QALYs** | **ICER** | **ICER Change** |
| **Base result** | | | | |
| DFLOT | 259010.78 | 3.89 | 124661.87 | - |
| FLOT | 154754.66 | 3.05 |  | - |
| **48.3% complete 10 postoperative cycles** | | | | |
| DFLOT | 248420.00 | 3.89 | 111998.19 | ↓ |
| FLOT | 154754.66 | 3.05 |  |  |
| **48.3% complete 8 postoperative cycles** | | | | |
| DFLOT | 237497.01 | 3.89 | 98937.27 | ↓ |
| FLOT | 154754.66 | 3.05 |  |  |
| **48.3% complete 6 postoperative cycles** | | | | |
| DFLOT | 226224.19 | 3.89 | 85458.06 | ↓ |
| FLOT | 154754.66 | 3.05 |  |  |
| **48.3% complete 4 postoperative cycles** | | | | |
| DFLOT | 214580.99 | 3.89 | 71535.96 | ↓ |
| FLOT | 154754.66 | 3.05 |  |  |
| **48.3% complete 2 postoperative cycles** | | | | |
| DFLOT | 202542.22 | 3.89 | 57140.88 | ↓ |
| FLOT | 154754.66 | 3.05 |  |  |
| QALYs; quality-adjusted life-year; ICER, Incremental Cost-Effectiveness Ratio; FLOT, fluorouracil + leucovorin + oxaliplatin + docetaxel; DFLOT, durvalumab + FLOT | | | | |

**Figure S1:** First-line treatment DFOLT survival curve.
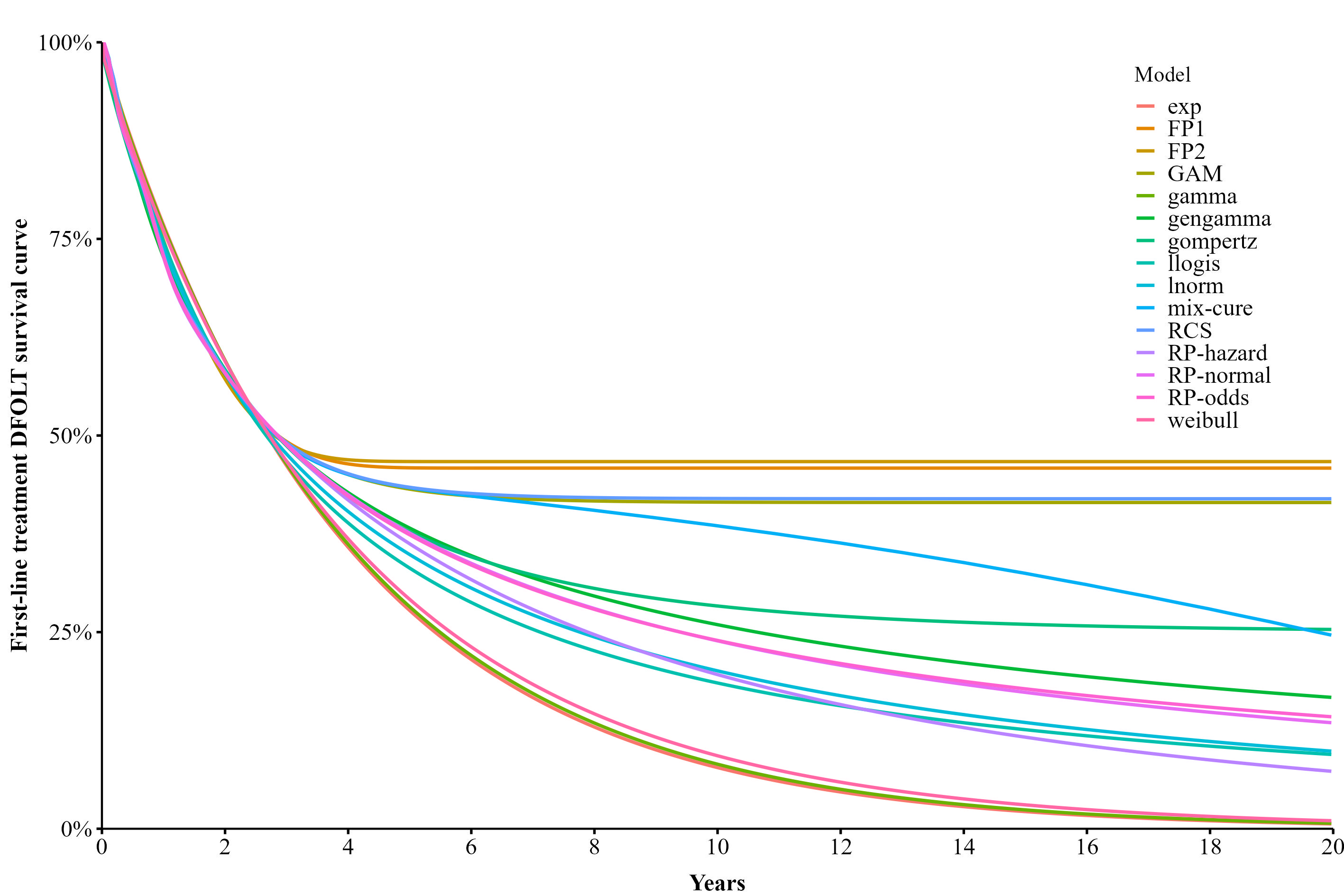


**Figure S2:** First-line treatment FOLT survival curve.**
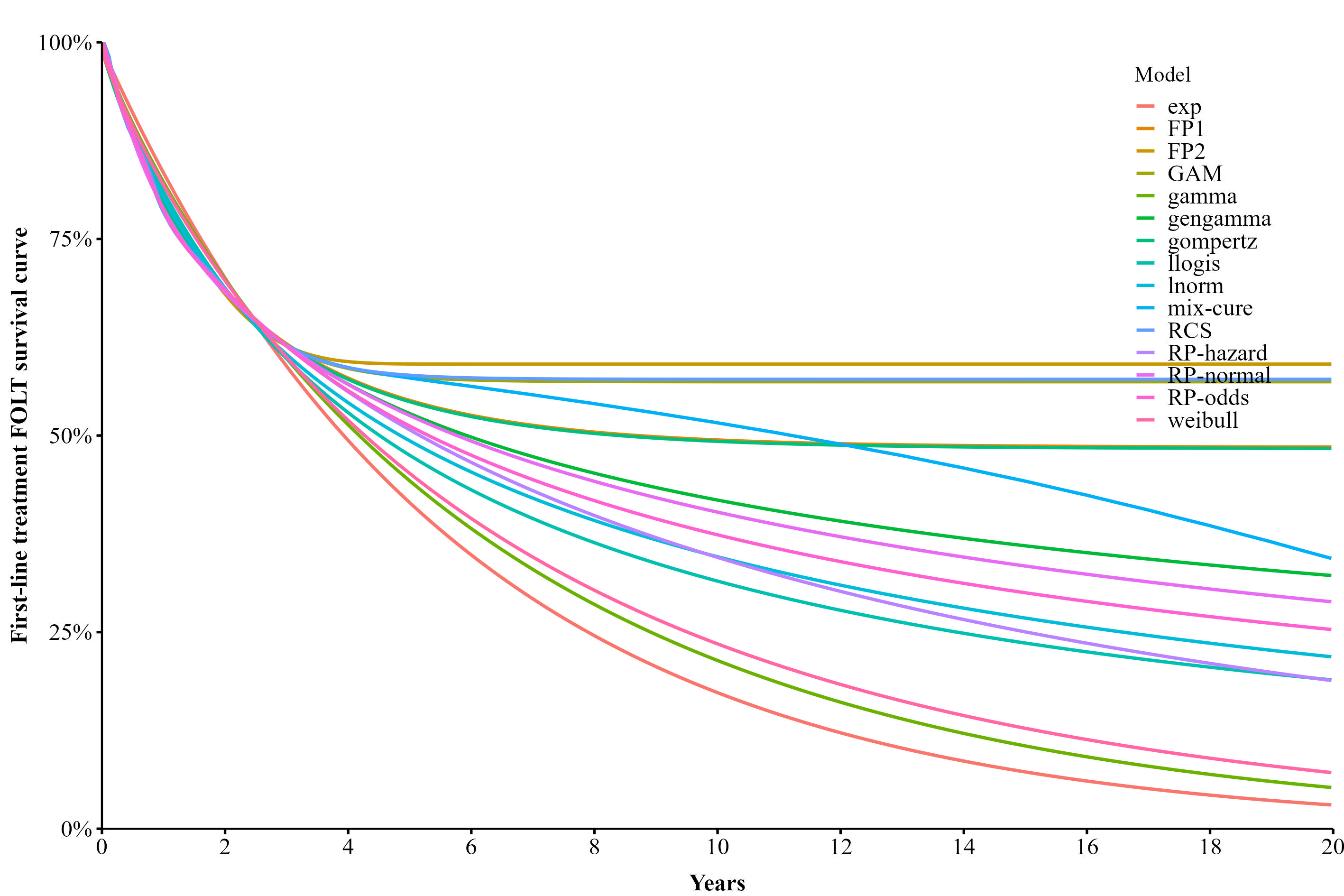
**

**Figure S3:** Second-line treatment OS survival curve.

**
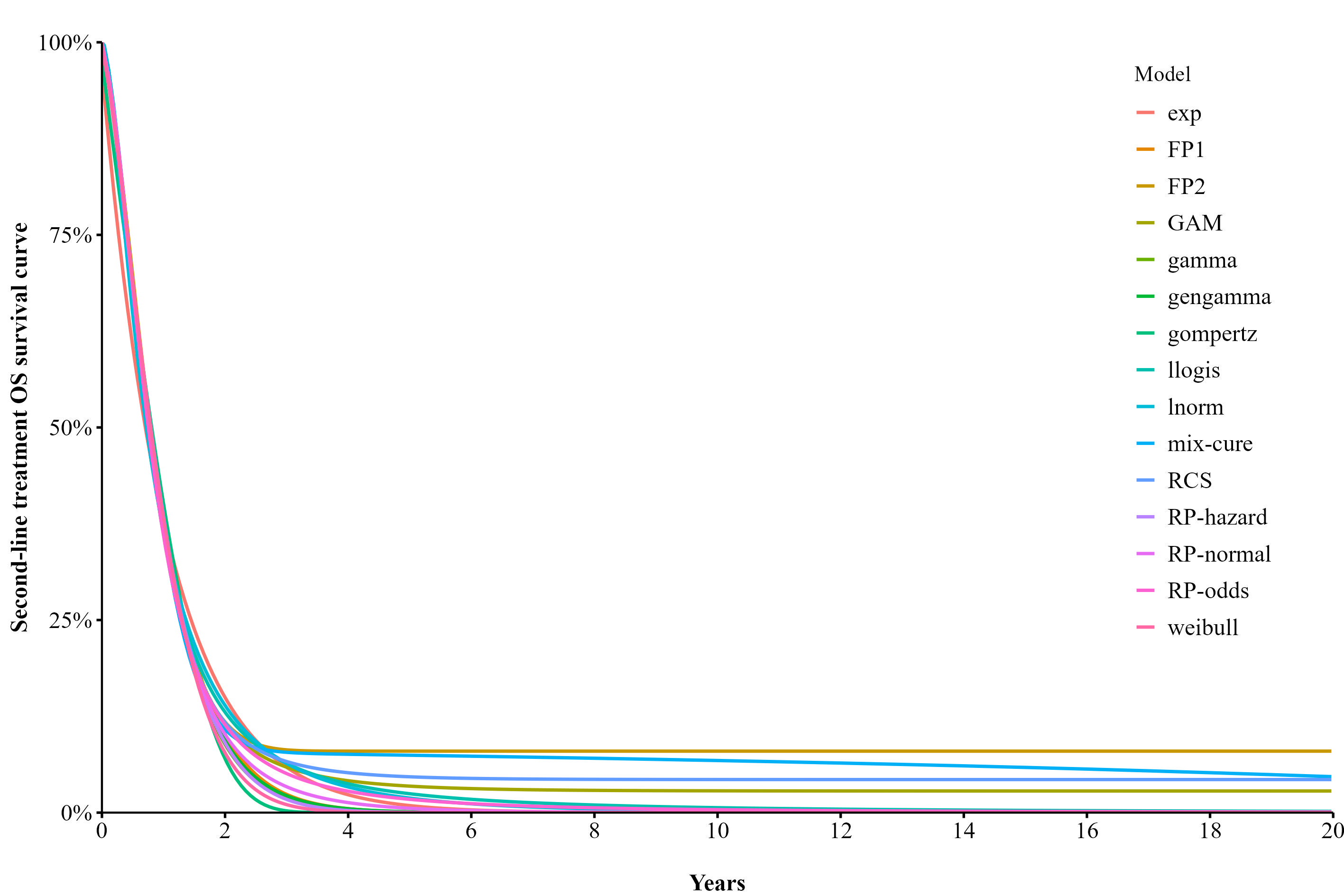
Figure S4:** Second-line treatment EFS survival curve.
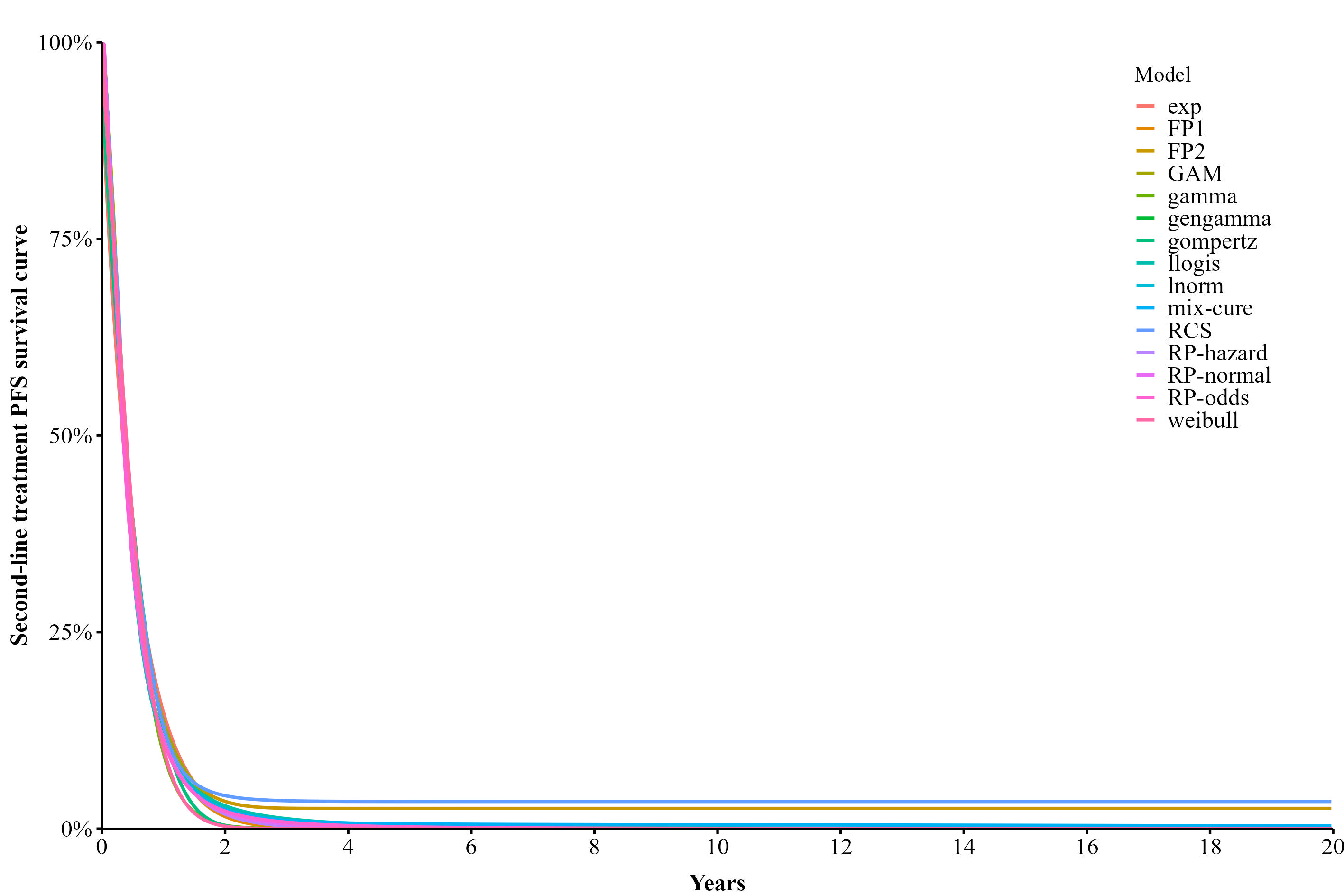


**Figure S5:** Estimated best-fitting first-line EFS curves for DFLOT and FLOT.


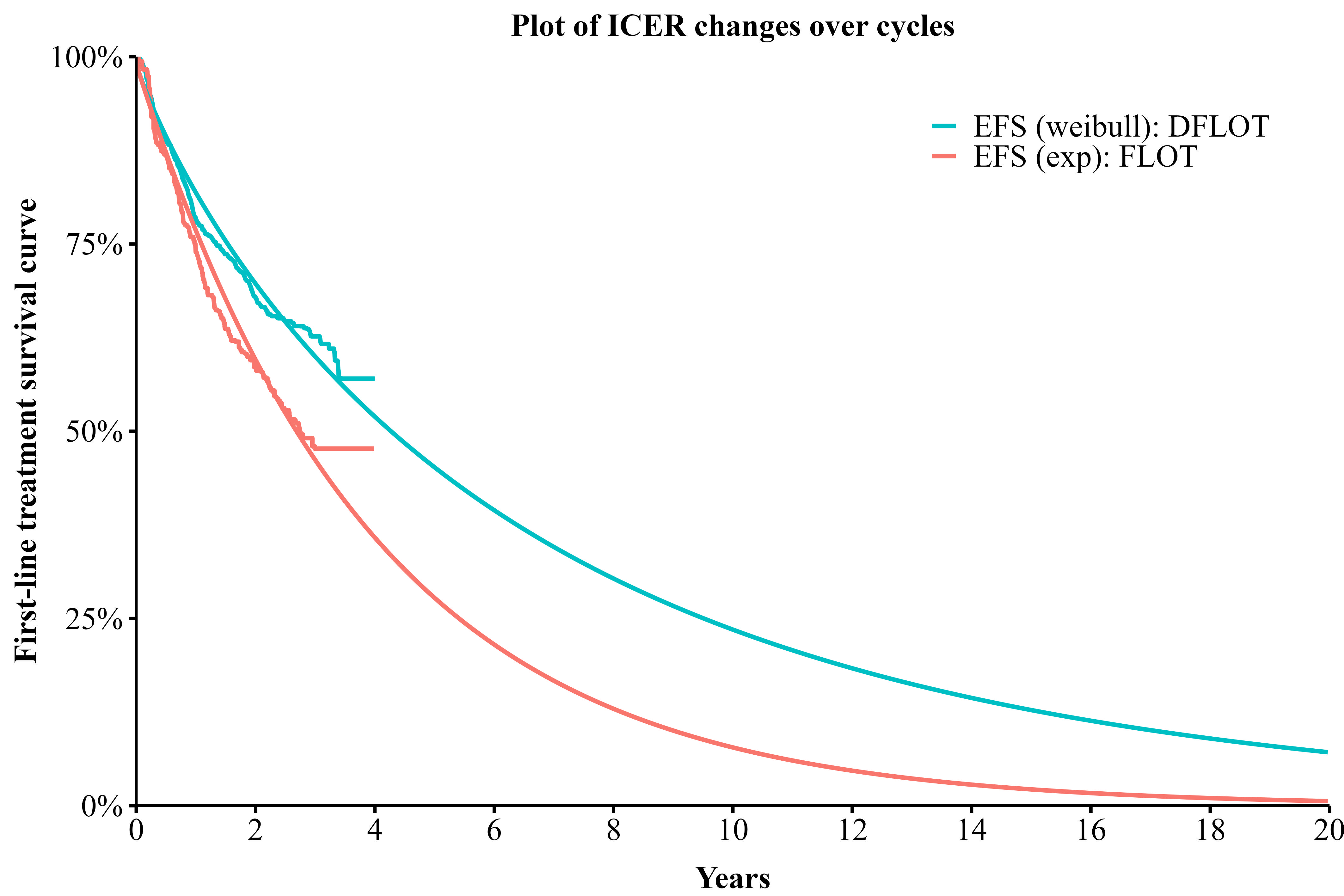


FLOT, fluorouracil + leucovorin + oxaliplatin + docetaxel; DFLOT, durvalumab + FLOT; EFS, event-free survival; OS, overall survival.

**Figure S6:** Estimated best-fitting for second-line treatment OS and EFS curves.


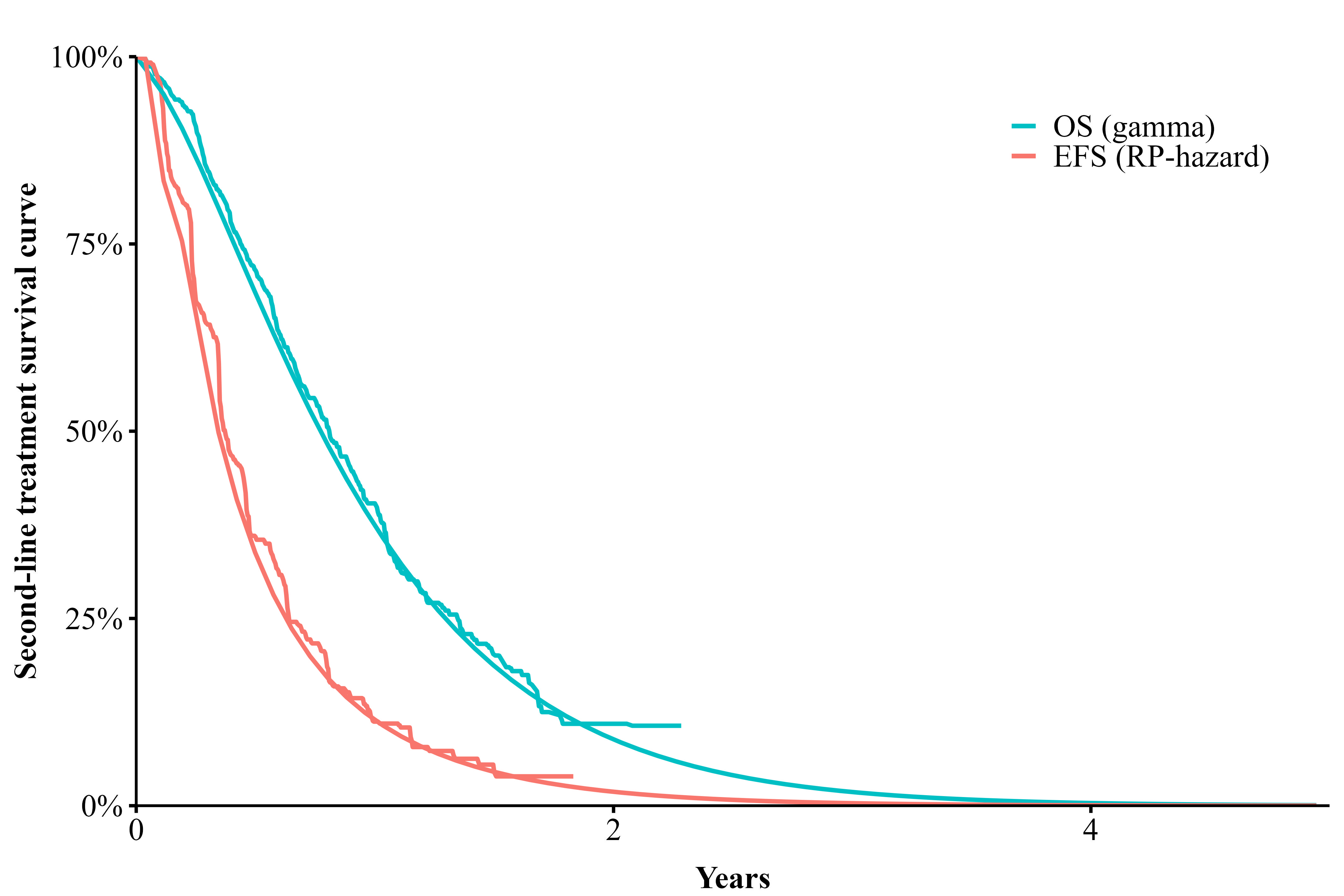


FLOT, fluorouracil + leucovorin + oxaliplatin + docetaxel; DFLOT, durvalumab + FLOT; EFS, event-free survival; OS, overall survival.
